# More than the ABCs: assessing the information needs of school nurses

**DOI:** 10.5195/jmla.2025.2137

**Published:** 2025-10-23

**Authors:** Annie “Nicky” Nickum, Karen Madura, Jeanne M. Link

**Affiliations:** 1 anicku2@uic.edu, Associate Professor and Information Services & Liaison Librarian, Library Liaison to the UIC Chicago Nursing Community, University of Illinois Chicago, Chicago, IL; 2 kmadur2@uic.edu, Clinical Assistant Professor, Department of Population Health, College of Nursing, University of Illinois Chicago, Chicago, IL; 3 Former Assistant Professor and Head Librarian, Information Services and Research, Library of the Health Sciences, MC 763, University of Illinois at Chicago, Chicago, IL

**Keywords:** School nurse, searching behavior, information literacy, Continuing Nursing Education

## Abstract

**Objectives:**

This interview study is a follow-up to a state-wide survey of school nurses' information needs conducted in 2022. Few studies have explored school nurses' information needs, with little focus on searching behaviors or barriers to practice.

**Methods:**

The principal investigator interviewed participants online about their thoughts on survey results, how they find information, and challenges within the profession.

**Results:**

After interviews with school nurses within rural, suburban, and urban districts in the state, the authors found that school nurses required information on finite topics but had little access to subscription resources, little training in critical analysis, and lacked time for professional development.

**Conclusion:**

School nurses within Illinois have routine information needs, most of which can be answered using a series of go-to resources. They are understaffed and overworked, which results in them having little time to do more than surface-level searching for care-related queries. Medical librarians may be able to assist this oft overlooked population with their information needs by providing workshops and resources.

## INTRODUCTION

School nurses influence student performance, absenteeism, and quality of life. As many as 25% of school-aged children in the United States may have a chronic condition, and students with health risks are more likely to struggle academically. [1]. The broad scope of school nursing and the complexity of issues addressed within a school community setting require advanced knowledge [2]. However, the preparation of school nurses varies widely across the country. For example, within Illinois, nurses fall into four categories (see Table 1): three of them are registered nurses (RNs) and the fourth are licensed practical nurses working under their [3].

**Table 1:** Tiers of School Nursing Practice in Illinois

Tier	Degree/Qualifications	Description
1	LPN (Licensed Practical Nurse or LVN (Licensed Vocational Nurse	Practice under RN supervision; limited responsibilities based on stipulations in their license.) 1 year program offered through vocational training schools or community colleges.
2	*RN (Registered Nurse) with ADN *BSN - Bachelors of Science in Nursing * Entry point for many nurses working in the school setting	ADN-RN-Associate degree in nursing – 2-year program licensed to practice per state requirements. BSN – 4-year degree. Additional training in community health, leadership and evidence-based practice
3	BSN with PEL-School Nurse Or Nationally Certified School Nurse	State certified school nurse - holds a professional educator license with an endorsement in school nursing (PEL-School Nurse) baccalaureate prepared nurses complete a school nurse certification program from an accredited institution of higher education and pass the state school nurse content exam. National Certification - formal recognition of advanced knowledge, competence and commitment to excellence in the specialty of school nursing.
4	MSN - Masters of Science in Nursing or DNP - Doctorate of Nursing Practice	Advanced degree with expertise in community health, advanced population health and or nursing education. Often working as a health services supervisor or nursing educator.

This lack of standardization makes it difficult to ensure nurses pursuing a career within school nursing receive equivalent levels of pre-professional education on skills like evidence-based practice and information literacy. The American Association of Colleges of Nursing in 2021 developed The Essentials: Core Competencies for Professional Nursing [4], which requires nursing curricula to address evidence-based practice and integration of evidence into nursing practice. While recent graduates may have benefitted from requirement, the majority of school nurses are not recent graduates who will not have benefitted from this curricula [5]. Moreover, even with the updated curricula, there is little focus within most EBP training programs on publicly available resources or critical appraisal of non-scholarly [6,7]; rather, much of the EBP content within nursing schools focused on licensed resources like CINAHL. These scholarly skills do not prepare school nurses to find publicly available evidence, nor to apply that knowledge pragmatically in the field.

Prior research into the information seeking behaviors of nurses affiliated with K-12 schools is limited; the majority of research on the information needs of nurses focuses on those employed by hospitals and healthcare systems [8–14]. Unlike nurses working in a healthcare system, nurses employed by school districts may have few options for accessing health information and are unlikely to have onsite support onsite from peer nurses or clinical librarians.[15]. In prior work, we found that school nurses generally consult both search engines and peers as sources for information[3]. We also found that while they search for information related to unfamiliar conditions, medications, and symptoms, they rarely use databases nor consult a librarian. This follow-up study sought to find the reasons and rationales behind the survey responses in order to develop an actionable plan for medical librarians to better support the school nursing population.

## METHODS

### Sample Selection

The previous study began in the fall of 2022 wherein the principal investigator sent out a request for participants through three professional listservs (Illinois Association of School Nurses, Illinois Department of Public Health Certified School Nurses, Illinois School Health Technical Assistance and Training) to recruit school nurses within the state of Illinois to answer questions pertaining to information seeking behaviors on the job. The final survey question asked whether participants would be interested in participating in follow-up studies. Those who indicated yes were contacted in September of 2023 and invited to participate in virtual one-on-one interviews to further examine the information needs of school nurses in the state. The research protocol was approved by the Illinois Institutional Review Board.

Compensation in the form of a $50 gift card was provided for interviewee participation. Due to the anonymity of the survey, prior data was not matched to the interviewees.

### Study Design

The previous survey captured demographic information such as number of years as a nurse as well as years working as a school nurse, education level, certification, school classification, grades served, and student population. The semi-structured interview questions were drawn from an earlier study of school nurses within a single metropolitan [16]. Interviewees were first asked about their thoughts on survey results, the remainder of the questions focused on searching behaviors, professional memberships and support, and challenges. All interviewees were emailed a handout prior to their scheduled interview (Appendix A) which contained the survey questions they previously answered, the results of the survey, and six core questions they would be asked during the interview. A complete list of questions including follow-ups is provided in Appendix B.

### Data Collection

The principal investigator completed all interviews virtually. Each meeting was conducted over Zoom in October of 2023, and each meeting was recorded and transcribed using Zoom features. Informed consent was obtained verbally, and interviewees were asked if they had any questions before each interview. The interviews consisted of the six questions plus additional follow-up questions (Appendix B). The interview lengths ranged from twenty to forty minutes. The investigator who conducted the interviews anonymized the data before it was reviewed by co-investigators. The audio recordings and anonymized transcripts were saved to a password-protected folder shared by investigators. Video recordings were not saved or shared.

### Data Analysis

Each investigator independently reviewed the transcripts. Inductive content analysis was used to identify keywords and phrases that represented themes across interviews [17]. Bimonthly meetings were held to discuss any differences and reach a consensus. Excel was used to organize the data. Transcriptions were reviewed continuously throughout the process and potential quotations identified as being representative of themes.

## RESULTS

### Overview

Of the forty-four respondents who took the initial survey and indicated an interest in further involvement, nineteen completed interviews. The interviewees represented multiple school districts across the state in rural, urban, and suburban areas. Their answers were categorized according to one of two overarching themes: information need or obstacles (Table 2).

**Table 2:** Themes and Definitions

**Information Need**	The information needed by school nurses to answer care-related questions.
*Types of Information*	The category of information needed - consumer health, policy, procedures, guidelines, and tertiary resources.
*Sources of Information*	The format and source of information - print, electronic, government, state, association, healthcare site, general website.
*Determining credibility*	How participants determined the information was credible and relevant to their information need.
**Barriers and Challenges**	The obstacles and challenges faced when attempting to answer a care-related question.
*Misconceptions*	The assumptions held by non-school nurses that affected the day-to-day work of school nurses.
*Workload*	The amount of work versus capacity of school nurses related to student ratios, staffing levels, and the nature of the work itself.
*Unforeseen Obstacles*	Obstacles in the school environment that were not foreseen by the school system such as the pandemic, influx of immigrants, budget cuts, and policy changes outside of the district.

### Reaction to survey results

The first interview question queried whether the given survey results were surprising or contained noteworthy information (included in their handout, Appendix A). Though some noted certain data points that surprised them such as low professional membership levels, interviewees were mostly of the opinion that the results were interesting but not surprising. Several noted that most of the survey respondents had been in the profession for at least ten years (82%). A nurse in an urban district thought the number of LPNs who responded (8 out of 405) was lower than expected. Three of the nurses expressed concern that only 30% of survey respondents were very confident in their awareness of current research in school nursing.

### Thematic Analysis

The interview questions were divided between searching behaviors and obstacles to obtaining information. Answers were categorized according to types of information needed, sources, and how credibility was determined. In addition to misconceptions, when school nurses spoke about barriers and challenges two themes emerged common for nurses in other settings: unforeseen obstacles and workload. Exemplars for each theme can be found in Appendix C.

### Information Needs

#### Types of Information

The majority of care-related questions that school nurses ask have concrete answers. Interviewees listed looking up medications and unfamiliar conditions as the predominant topics of information searching. This was followed by school health policies and procedures. School nurses are tasked with staying on top of immunization compliance, and the public health of the school. They may need to educate teachers on how to best monitor a medically fragile student or connect a new student's family with community resources. Students who undergo hospital procedures receive discharge instructions for them and their families. While they often follow up with either a hospital team or primary care, there may be little or no inclusion of the school nurse.

I know what I would do in the hospital, but here it's like I'm looking up after the fact of what else could we do for him while he's here for immediate treatment. He doesn't come with a healthcare plan. We have not been able to get ahold of doctors.

For instance, I had a student who had a new heart procedure done, and I could not find any information on it at all. I ended up contacting the doctor's office to get what information they had on it. So just that I knew what had happened. I mean, I know the outcome, but just to know for Post OP. What to be looking for so definitely, new medical information is hard to find.

#### Sources of Information

The majority of interviewees (14/19) stated they used preferred websites and search engines to find answers to care-related questions. Frequently used sites included the Center for Disease Control (CDC), the state health department, and well-known institutions such as the Mayo and Cleveland Clinics. One interviewee mentioned a text, the American Academy of Pediatrics' *Managing Infectious Diseases in Childcare and* [18]; she always kept it on hand. Many said if they were unsure, they found a way to ask peers or they would call the student's physician in the case of follow-up care. None of the participants considered the library as a potential resource for information. It was additionally mentioned that credible sites such as the National Institute of Health were learned about during graduate studies and never mentioned as part of initial nursing education.

#### Evaluation of Information

When asked about how answers to care-related questions were deemed credible, answers fell into three categories: information was correct based on experience, information was obtained from a site perceived to be credible, and consensus across multiple sites. Resources perceived to be credible were ones from associations, top institutions, and government funded. Once credible sites were identified, they became preferred resources. As one nurse put it, “There's a disconnect. You learn in school that evidence-based practice is king, but you don't learn how to apply it to practice.”

### Barriers and Challenges to Information Seeking

#### Lack of Understanding

Interviewees felt that the role of the school nurse is not fully understood by school administrators and staff. This lack of understanding of their role can lead to budget cuts, not being kept in the loop with other student services providers such as the school psychologist and social worker, not being on the Wellness Committee or being involved in decisions that affected student health, and administration not understanding their role or priorities. Interviewees spoke of being thought of only in terms of first aid; that other staff would see them on the phone or on a computer when their office was empty and not realize they were contacting doctors and updating student information.

In advocating for things, you have to fight twice as hard for anything. Right?... So, this year they decided to cut our budget… The counselors still get their money, and the psych still get their money, but we'll take money from the nurses.

Further complicating the issue is the idea that school nurses do not require advanced skills because their work is only surface level.

Because if your perception of someone is that they're handing out, you know, first aid, why would they need access to evidence based practice research? That's not within their scope, if that's your understanding of it.

#### Workload

The nurses spoke of their heavy workloads. While some schools had multiple school nurses or a school nurse and aides working under them, many had a single nurse; there was no one on site for second opinions, no one to fill in if they were absent, and lack of inclusion in staff meetings. The model might work in the ideal situation of the occasional student coming in for a basic first aid and minor illnesses, but it's hardly suitable for when multiple students need care or there's an emergency. Nearly all said they were too busy in the course of the day to do any sort of research; they said they did not have the capacity to devote themselves to an in-depth search of resources because of their workload and potential for interruption. Having a single nurse in the school presents certain dangers;

Biggest challenge is really no one in the school setting understanding your scope of practice and usually being the only medical professional around right now, personally, I have 3 schools that I serve independently. I get there at 7:30 leave at 3:30. I don't get a bathroom break. I don't get a lunch break I'm kind of just everywhere all at once. If I'm in the middle of a procedure, I can't leave the procedure. Then we're having to utilize 911 because I'm in the middle of a urinary cath(eter). I just can't leave the cath in and then run procedure. So, it's a lot of just trying to get them to understand how dangerous that is. So that's my biggest thing right now is fearing for my license.

Other participants noted the lack of time for research. One nurse said: “I mean definitely have convenient access. Don't always have time to look things up and do any research. Mostly we're doing that on our own time. Right?”

There was no way I could do that during the day. So, if I was looking into things, it was always on my own time or in the evening or on the weekends or I would stay late when it was quiet. Now with my position, I kind of go all over the district.... It's super time consuming and if you are one nurse in a building there's just no way like you have time to do that. You're doing it in the summer when you're not getting paid or you're doing it like after hours.

#### Unforeseen Obstacles

This lack of understanding of the school nurses' responsibilities and their workload, makes unforeseen issues all the worse. For example, schools did not plan for the mental health needs of students during or after the pandemic.

I think that has really ramped up (student mental health issues). You know, the kids don't know the difference between I don't feel well because I'm sick, and I don't feel well because I'm depressed or I'm scared, or I'm anxious. They just don't know. So, the volume of students we're seeing has just skyrocketed all around our district…… We don't have time to look into things. So, I think that's part of the problem. But, I guess, knowing more about where they're coming from, what they were dealing with would help too.

Nurses have limited ability to influence the system-wide changes needed to fully address these kinds of issues. Yet, it is this kind of community-specific information that cannot be found on general websites or with basic search skills.

## DISCUSSION

The results of this study underline the importance of school nurses and the need for them to have training in answering health-related questions at the point of care using publicly available resources online. School nurses within Illinois are highly educated and invested in the health and wellbeing of their students yet often do not have the resources for in-depth research to support student health. School-aged students in the United States present with increasingly complex care needs, which necessitate an informed nursing workforce. Meeting this challenge is difficult when a single school nurse is the sole healthcare provider at the school or even for the entire district [19].

Challenges unique to school nurses appear to be systemic, related to the education aspect of their role. The information they use to care for students must be “current, accessible, and useful,”[20], but multiple barriers to reliable online medical resources persist, including a lack of time to perform careful searches for information, a lack of knowledge about technology, and a lack of administrative support or understanding of the nurse's role, evident by a lack of access to health science databases [20]. These constraints lead to search engines being used rather than databases, and a deficit in patient education resources in other languages [20–22]. These findings are consistent with studies in Tulsa, [16] and rural Washington [21]. That such similar findings were made in such different environments several years apart speaks to the ongoing severity of the issue.

In an inpatient setting, the work of a nurse is well-defined, and other healthcare practitioners understand the basics of the role and associated responsibilities. This is not the case in schools [23]. The lack of understanding of their role meant budget cuts, not being kept in the loop with other healthcare providers such as the school psychologist and social worker, not being on the Wellness Committee or being involved in decisions that affected student health, and administration not understanding their role or priorities. The interviewees spoke of being thought of only in terms of first aid; other staff would see them on the phone or on a computer when their office was empty and not realize they were contacting doctors and updating student information.

1. There is a vast difference between the educational levels of nurses who practice in the school setting. These differences in education and experience in the specialty influence the nurses' information needs and access to evidence-based resources. It is well documented that nurses across the board do not implement evidence-based practice [24]. School nurses are using search engines to answer care-related questions to determine best practice for taking care of children. More straightforward questions, such as background information about unfamiliar conditions or side effects of medication, can be found on sites such as state health departments, the CDC, and other credible institutions. More alarming is the idea that they lack access to information about current advances in healthcare, such as new methods in disease management. The results point out that there is a need for professional development to enhance their information literacy skills and awareness of freely available resources. School nurses, while generally satisfied with their ability to find information, declare interest in attending workshops to improve their skills. Due to the paucity of research on the information needs of school nurses, it is imperative to understand how they use information before any education or interventions are designed [3,16].

The interviewees identified several barriers to seeking information. Many overlapped with their peers practicing in hospitals – lack of time, not part of the workflow, and insufficient training [25]. Problems unique to school nursing related to schools' focus on education rather than on healthcare: the needs of teachers prioritized over school nurses, administrators not understanding the scope of practice, practice guidelines not anticipating application outside the inpatient environment, and school policies not being informed by healthcare personnel.

The challenges of staffing and support led to the lack of advocacy for the profession, administration not prioritizing school nurses, and not enough hours in the day to get work done. Regarding specific populations, the wide breadth of knowledge needed to work with students ranging in age from elementary to high school, those working with students of lower socioeconomic status spoke of families needing community support, immigrant students without a translator for healthcare needs, navigating how to best care for transgender students, and lack of support for nontraditional schools such as private or population specific.

Several interviewees lamented that they felt unprepared to navigate caring for refugee students. The complicated matter of refugee children may have been covered at the state or district level, but no plan can cover all the necessary details such as lack of health records or family communication. The nurse went on to explain that there had been an influx of refugee students and only one teacher in the school was able to translate: an inappropriate workaround as far as patient privacy and the teacher's own workload. As the nurse is not the only staff member impacted by language barriers, school administrators should try to identify and implement a cost-effective solution for English-language learners.

## LIMITATIONS AND NEXT STEPS

The initial survey captured the opinions of school nurses who were members of one of three statewide listservs and the interviewees were a subset; sampling was self-selective. Demographic information was captured for the initial survey participants but no data apart from district type and gender were collected from interviewees. Despite the questions' focus on information access and retrieval, the interviewees spoke most vehemently about lack of understanding on their role and challenges to the profession. As with any semi-structured interview, the need for more nuanced information was not discovered until the analysis phase; future research will address these gaps.

This was the second of a three-part study on school nurses. Data analysis of both the survey and interviews revealed gaps in questions and a need for more nuanced information. To that end, a new survey was developed to address these knowledge gaps. In summer 2024 it was distributed nationwide along with a URL for a library guide, created by academic librarians, featuring links to credible health information resources without a paywall.

Some of the survey questions aim to gather feedback and opinions about the library guide. Do participants find it relevant and potentially useful to the profession? Ideally this is an initial step towards the current end goal – development of a free, self-paced online continuing education course designed expressly to address the information needs of practicing school nurse in the United States.

### Recommendations

An obvious resource for any professional is a relevant association for their discipline. The National Association of School Nurses website is robust as it provides open access publication, toolkits, and continuing education. Unfortunately, some of its content is only available to members, leaving school nurses in many districts, whether because the pay is low or because districts will not pay professional dues, at a distinct disadvantage.

Librarians meet patrons at the point of need and pride themselves on getting the right information to the right person at the right time. A public library may be able to help and, depending on the city, may even have access to several databases, but they rarely have training to handle healthcare-related queries [26]. School nurses may feel confident in their searching abilities, but information on the general internet or in print resources may be dated; it may be difficult to find resources to assist a student who underwent a newer procedure or is using a recently developed device such as one used for glucose monitoring.

Medical librarians have the potential to address school nurses' gaps in knowledge. Librarians are often involved in teaching research to nursing students, and more effort could be made to include resources common to public health and the overall development of online search skills. Regarding school nurses already in practice, outreach can be done to local school districts and state school nurse consultants for professional development. The first step is awareness on both fronts; librarians become aware of the unique information needs of school nurses and school nurses, for their part, become aware that help is available.

## CONCLUSION

Like their counterparts in healthcare institutions, school nurses have limited time to conduct in-depth research as they barely have the time to look up information at all. Due to the autonomous nature of their work, it is imperative they have the training and resources to answer clinical or care-related questions. School nurses do not have a designated information professional to whom they can turn when stuck with a care-related or clinical question.

Medical librarians have the potential to address school nurses' gaps in knowledge. Librarians are often involved in teaching research to nursing students, and more effort could be made to include resources common to public health and the overall development of online search skills. Regarding school nurses already in practice, outreach can be done to local school districts and state school nurse consultants for professional development. The first step is awareness on both fronts; librarians become aware of the unique information needs of school nurses and school nurses, for their part, become aware that help is available.

## Data Availability

Data associated with this article are available on the authors' institutional repository at the following URL: https://indigo.uic.edu/articles/dataset/Codebook_for_interview_themes/26332339.
