# Supplementary material for: More than the ABCs: assessing the information needs of school nurses
Source: J Med Libr Assoc. 2025 Oct 23;113(4):310–7. doi: 10.5195/jmla.2025.2137 (PMC12604073; doi:10.5195/jmla.2025.2137)
Supplement: Supplementary file 1 — Appendix A [file jmla-113-4-310-s01.docx]

Interviewee Handout

Thank you for scheduling your interview to discuss the information needs of school nurses within Illinois! This handout includes all the questions in the survey in addition to a graphical representation of the results. Take a few minutes to look over this handout, making note of anything you find interesting or surprising. A list of questions that will be asked during the interview itself is at the end. Please note that this is not a complete list of questions; there may be follow-up questions depending on your responses.

**Survey Questions**

1. How often do you search for each of the following types of information as part of your school nursing responsibilities?

Never, less than once a month, monthly, more than once a month, weekly, more than once a week

*Health conditions, illnesses or injuries*

*Symptoms*

*Treatments*

*Prescriptions or medications*

*Immunization schedules*

*Physicians or healthcare facilities*

*State health policies or laws*

*School or education policies or laws*

*Clinical guidelines*

Please note any other type(s) of information you frequently search for

________________________________________________________________

1. How often do you consult each of the following sources for information as of your school nursing responsibilities?

Never, less than once a month, monthly, more than once a month, weekly, more than once a week

*Search engines (Google, Bing, etc.)*

*Health websites (Healthline, Livestrong)*

*Social media sites or apps*

*National or Illinois School Nurse Association site*

*Government and district websites (local, state, federal; CDC, public health department)*

*Nursing or medical textbooks*

*Nursing or medical journals or magazines*

*Databases (PubMed, CINAHL, Medline)*

*Fellow nurses*

*Libraries or librarians*

1. Please note examples of health websites and any other source(s) you frequently consult for information

________________________________________________________________

1. Do you have access to paid resources such as CINAHL, ERIC, or PsycInfo? If so, please list

- Yes ________________________________________________
- No
- Unsure

1. Please indicate your perceptions regarding the following statement as they pertain to your current position and school environment.

Strongly disagree (1), somewhat disagree (2), neither agree nor disagree (3), somewhat agree (4), or strongly agree (5).

*I am satisfied with my ability to search for information necessary to perform my school nursing responsibilities*

*I have convenient access to information sources needed to perform my school nursing responsibilities*

*I am aware of current research related to school nursing practices*

*I would be interested in attending a class, workshop, or online module regarding access to health information*

*I have convenient access to the internet while at school*

1. In the space below, please describe any school health initiatives (such as health awareness campaigns, screenings, or education) in which you are involved.

________________________________________________________________

1. Please indicate you highest level of preparation to practice nursing in Illinois schools

- I am a non-certified AND-RN
- I am a non-certified BSN-RN
- I have an Illinois Professional Educator License - Certified School Nurse (PEL-CSN)

1. What is your highest level of education?

- LPN
- ADN
- BSN
- MSN
- MEd
- DNP, PhD

1. How many years of experience do you have as a nurse? Select one

- 0-2
- 3-4
- 5-9
- 10 or more

1. How many years of experience do you have as a school nurse? Select one

- 0-2
- 3-4
- 5-9
- 10 or more

1. Please select the organizations in which you are a member
2. National Association of School Nurses
3. Illinois Association of School Nurses
4. Neither
5. Select the appropriate level of school that you oversee (choose all that apply):
6. Early childhood (ages 3-5)
7. Elementary (K-5)
8. Middle school (6-8)
9. Combined Lower (K-8)
10. Combined Upper (7-12)
11. High school (9-12)
12. Other, please describe ________________________________________________
13. How would you describe your school district?

- Rural (1)
- Suburban (2)
- Urban (3)

1. Estimate the number of students for whom you are responsible

________________________________________________________________

1. Estimate the number of those students with special healthcare needs

________________________________________________________________

**Survey Results**

Table 1: Demographics of school nurses in Illinois

| **Highest level of certification** | | | 398 |
| --- | --- | --- | --- |
| Non-certified ADN-RN | 43 | 11% |  |
| Non-certified BSN-RN | 142 | 36% |  |
| Professional Educator License - Certified School Nurse | 213 | 54% |  |
| **Education Level** | |  | 401 |
| LPN | 8 | 2% |  |
| Assoc | 44 | 11% |  |
| BSN | 239 | 60% |  |
| MSN | 72 | 18% |  |
| Med | 30 | 7% |  |
| DNP/PhD | 8 | 2% |  |
| **Years of experience as an RN** | | | 403 |
| 0 to 2 | 8 | 2% |  |
| 3 to 4 | 16 | 4% |  |
| 5 to 9 | 50 | 12% |  |
| 10 or more years | 329 | 82% |  |
| **Years of experience as a school nurse** | | | 403 |
| 0 to 2 | 65 | 16% |  |
| 3 to 4 | 58 | 14% |  |
| 5 to 9 | 127 | 32% |  |
| 10 or more years | 153 | 38% |  |
| **School levels overseen*** | |  | 403 |
| Early childhood | 148 | 37% |  |
| Elementary | 196 | 49% |  |
| PreK-5th* | 114 | 28% |  |
| Middle school | 133 | 33% |  |
| Combined lower (K-8) | 93 | 23% |  |
| Combined Upper (7-12) | 40 | 10% |  |
| High school | 142 | 35% |  |
| Other | 47 | 12% |  |
| **District type** | |  | 395 |
| Rural | 63 | 16% |  |
| Surburban | 251 | 64% |  |
| Urban | 81 | 21% |  |
| **Membership in professional organizations*** | | | 403 |
| NASN | 228 | 57% |  |
| IASN | 210 | 52% |  |
| APHA | 6 | 1% |  |
| APHN | 2 | 0% |  |
| none | 151 | 37% |  |

*Indicates more than one response could be selected

Table 2: Top school health initiatives

| Top Ten Initiatives |
| --- |
| 1. Vision and hearing screenings |
| 2. Mobile dentist |
| 3. Immunization screening and clinics |
| 4. Hygiene and cough etiquette |
| 5. Staff education |
| 6. Health education |
| 7. Teaching CPR/AED classes |
| 8. Sexual health and development |
| 9. Wellness committee |
| 10. Implementing national programs |

Figure 1: Number of students per nurse assignment


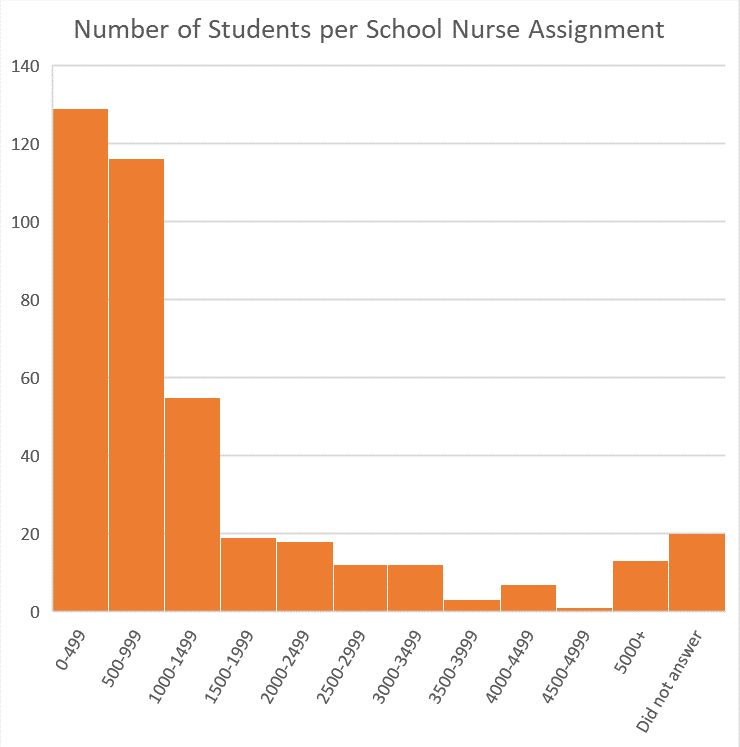


Figure 2: Percentage of students with special healthcare needs


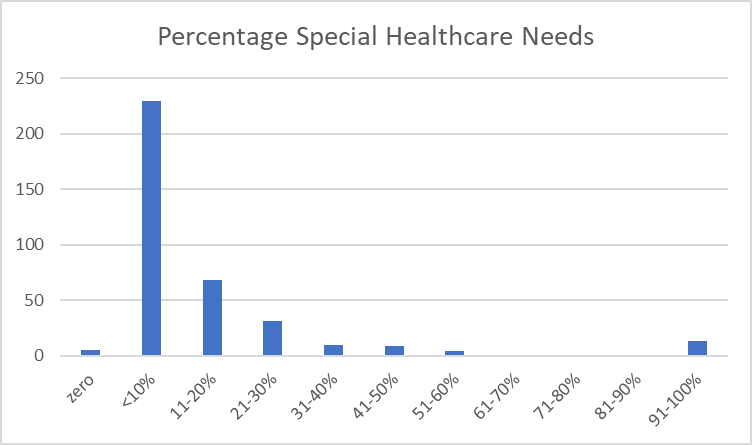


Figure 3: Frequency of topics searched by school nurses


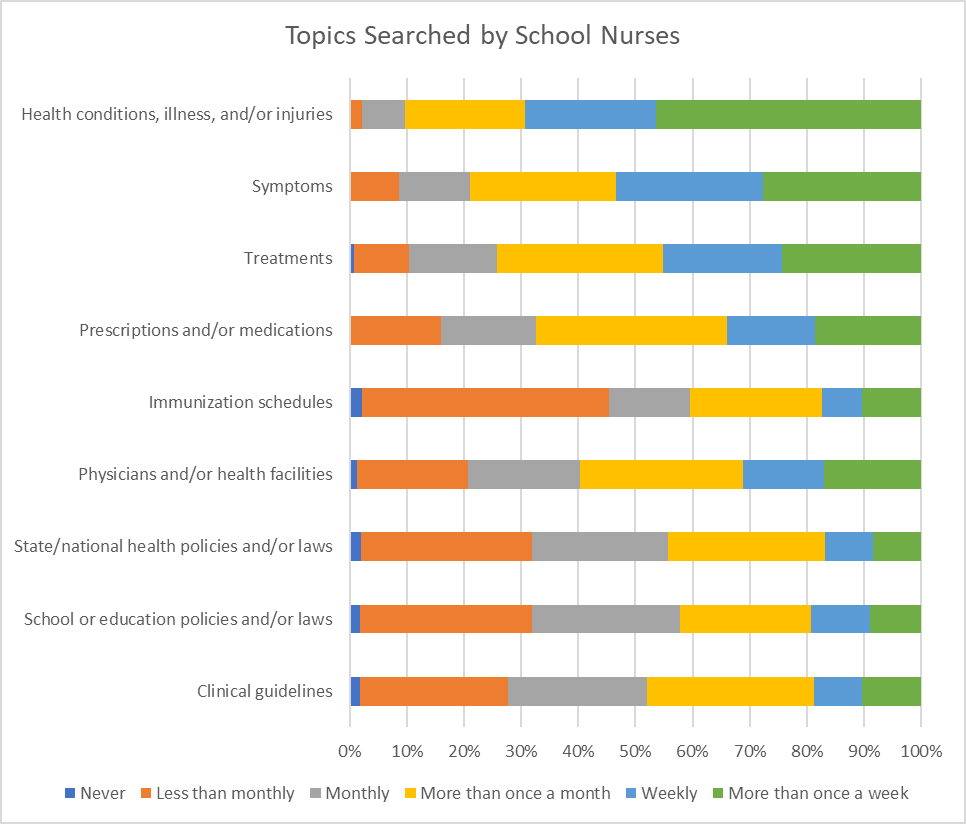


Figure 4: Sources of information


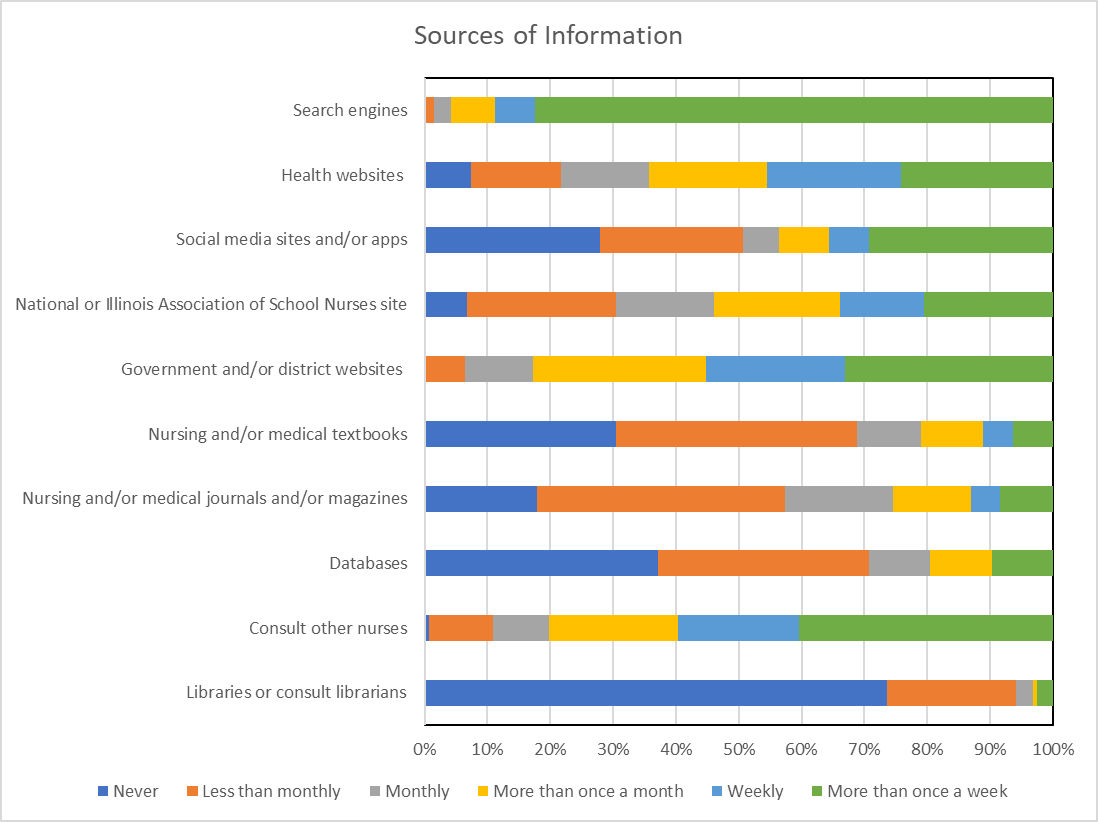


Figure 5: Sources of information by educational level


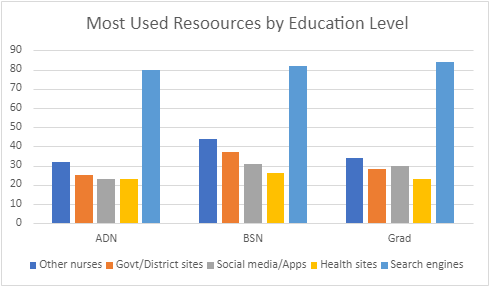


Figure 6: Perceptions of searching ability


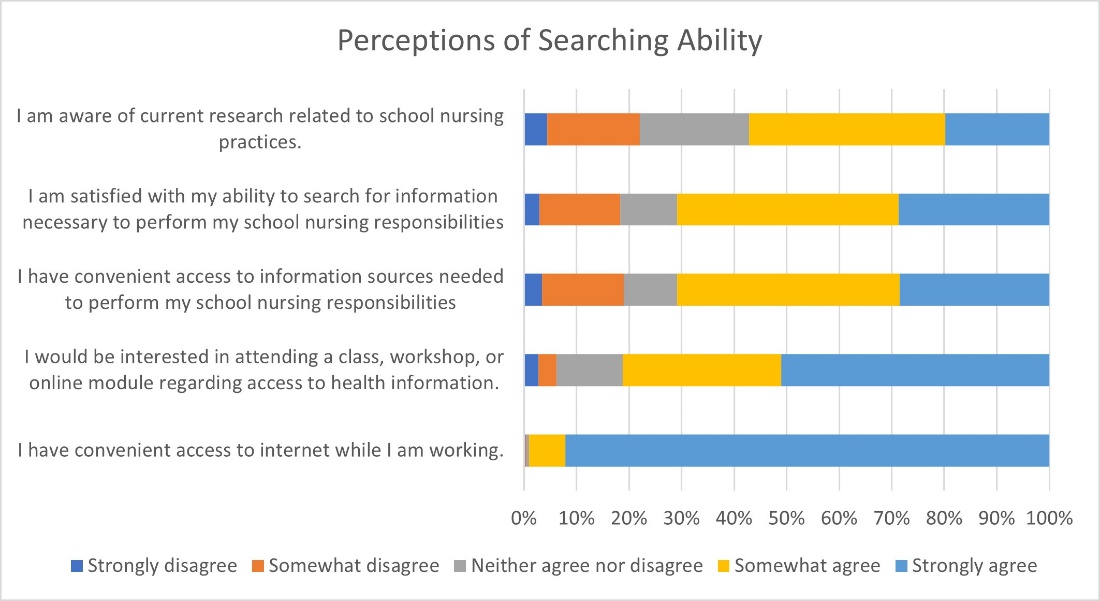


**Interview Questions**

1. We’ll begin by looking at the results of the survey that you all took last fall. You have been given a handout including all the questions in the survey in addition to a graphical representation of the results. Take a few minutes to look over this handout, making note of anything you find interesting or surprising.
2. Let’s now start to think about your own searching behavior. Starting with broad generalities, please think about your ‘typical’ day in the school nurse office. Describe a type of information that you frequently search for to perform your daily responsibilities.
3. Now, moving from the broad to specific, can you describe a time when you needed to search for information? What was the situation and what piece of information did you need?
4. Thinking back on the survey that you took last fall, you were asked to describe school health initiatives in which you were involved or of which you are a part. Table 2 lists the most commonly described health initiatives from the survey. Do you feel this is an accurate representation? If not, what is missing?
5. Looking at Table 1 regarding demographics of school nurses in Illinois. Based on your experience, is this an accurate representation? Why or why not? Are you a member of any professional organizations?
6. To close this session, what are some of the biggest challenges that you face as a school nurse (for example lack of time, isolation from medical peers)?
