# Supplementary material for: More than the ABCs: assessing the information needs of school nurses
Source: J Med Libr Assoc. 2025 Oct 23;113(4):310–7. doi: 10.5195/jmla.2025.2137 (PMC12604073; doi:10.5195/jmla.2025.2137)
Supplement: Supplementary file 2 — Appendix B [file jmla-113-4-310-s02.docx]

Appendix B: Interview Questions

1. We’ll begin by looking at the results of the survey that you all took last fall. You have been given a handout including all the questions in the survey in addition to a graphical representation of the results. Take a few minutes to look over this handout, making note of anything you find interesting or surprising.

1. Follow-up question: What do you find interesting about these results? Looking specifically at Figure X, do you feel that these levels of perceptions are accurate? If not, what do you feel is misrepresented?

1. Let’s now start to think about your own searching behavior. Starting in broad generalities, please think about your ‘typical’ day in the school nurse office. Describe a type of information that you frequently search for to perform your daily responsibilities.

1. Follow-up question: What are sources that you generally use to find needed information? Have you consulted databases like PubMed or websites like MedlinePlus? Why or why not? In a typical Google search, how do you determine the quality and accuracy of the sites that you find?

1. Now, moving from the broad to specific, can you describe a time when you needed to search for information? What was the situation and what piece of information did you need?

1. Follow-up question: What did you do? How long did your search take you? Were you content/satisfied with the quality of the information you found? Did you consult anyone else?

1. Thinking back on the survey that you took last fall, you were asked to describe school health initiatives in which you were involved or of which you are a part. This screen lists the most commonly described health initiatives from the survey. Do you feel this is an accurate representation? If not, what is missing?

1. Follow-up question: What is your role in these initiatives? How could you feel better supported in this role?

1. Looking at Table 1 regarding membership in a school nurse association, only about half of the survey participants indicated membership. Are you a member in an association? If so, which association?

1. Follow-up: How do these professional associations support your work?

1. To close this session, what are some of the biggest challenges that you face as a school nurse (for example lack of time, isolation from medical peers)?

1. Follow-up question: Do these challenges affect your ability to get access to information you need? Follow-up question: Do these challenges affect your ability to get access to information you need? Thinking back to some of the services I mentioned our library providing and resources that I overviewed, can you envision ways that our library might be able to help address these challenges?
