# Supplementary material for: More than the ABCs: assessing the information needs of school nurses
Source: J Med Libr Assoc. 2025 Oct 23;113(4):310–7. doi: 10.5195/jmla.2025.2137 (PMC12604073; doi:10.5195/jmla.2025.2137)
Supplement: Supplementary file 3 — Appendix C [file jmla-113-4-310-s03.docx]

Appendix B: Themes and Exemplars

| Theme | Exemplar |
| --- | --- |
| **Information Need** | |
| *Types of Information* | We have an influx of students. There are transfer students or refugee students and there's language barriers. So, a lot of our time is spent on education, but also finding resources for students, like finding mental health resources. |
| *Sources of Information* | There's like 2 medical websites that I use. I don't use anything other than those 2 that I know are legit medical websites. I always look for those on the search engine. |
| *Determining credibility* | Don't give much thought to that [determining credibility]. To be honest, I kind of rely on my past experience. Yeah, I don't know how a new nurse would do that, but I haven't really given that much thought. |
| **Barriers and Challenges** | |
| *Misconceptions* | I would say one of the biggest challenges is very often people don't know what we do. I think the perception of school nursing is we sit in an office, and we read romance novels and handout band-aides and ice packs. And I think a lot of the onus for that perception lies with us, because historically, we, as a nursing profession don't stand up and speak for ourselves, and I think that's probably doubly true for school nursing. Because if your perception of someone is that they're handing out first aid, why would they need access to evidence based practice research? That's not within their scope, if that's your understanding of it. |
| *Workload* | There was no way I could do that [research] during the day. So, if I was looking into things, it was always on my own time or in the evening or on the weekends or I would stay late when it was quiet. Now with my position, I kind of go all over the district. It's still hard to find time to lock yourself in a room and do a review because you need peace and you need quiet to be able to focus. That’s part of the problem is the constant interruptions why it takes so long because you know you're constantly on. |
| *Unforeseen Obstacles* | I'm researching all the immunizations for students... I'm in a bilingual school that has a dual language program and a lot of bilingual families. So, we get a lot of students that are brand new to the country, so I do fairly often receive immunization records from out of the country. So, I do a lot of research on those like which immunizations will transfer over if they're given in a different country, because some of the formularies are different. So, they don't necessarily meet our state requirements for exclusion. |
